# Cuttlefish ink nanoparticle-engineered hydrogel microspheres synergistically attenuate disc degeneration via antioxidant defense and matrix synthesis activation

**DOI:** 10.1016/j.mtbio.2025.102244

**Published:** 2025-08-25

**Authors:** Chenyang Jin, Jianan Chen, Yan Miao, Xin Tian, Jia Wang, Fan He, Yijie Liu, Yong Xu

**Affiliations:** a Department of Orthopaedics, The First Affiliated Hospital of Soochow University, MOE Key Laboratory of Geriatric Diseases and Immunology, Suzhou Medical College, Soochow University, Suzhou, 215000, Jiangsu, China; b Orthopaedic Institute, Suzhou Medical College, Soochow University, Suzhou, 215000, China; c Department of Orthopaedic Surgery, The Fourth Affiliated Hospital of Soochow University, Suzhou Medical College, Soochow University, Suzhou, 215000, China

**Keywords:** Hydrogel microspheres, Nucleus pulposus regeneration, Cuttlefish ink nanoparticles, Antioxidation, Extracellular matrix

## Abstract

Intervertebral disc degeneration (IVDD) is a leading cause of spinal disorders, affecting millions globally, particularly the aging population. Current treatments, however, fail to fully restore disc structure and function, highlighting the need for regenerative therapies. This study aims to construct an antioxidant artificial nucleus pulposus (NP) by incorporating cuttlefish ink nanoparticles (CINPs) into GelMA microspheres, thereby enhancing nucleus pulposus cell (NPC) viability and extracellular matrix (ECM) synthesis. Oxidative stress is a key driver of disc degeneration. CINPs, rich in proline and fucose, significantly enhanced the antioxidant capacity of NPCs, as evidenced by reduced intracellular reactive oxygen species (ROS) levels and activation of the nuclear factor erythroid 2-related factor 2/heme oxygenase-1 (NRF2/HO-1) pathway in our study. In vitro experiments demonstrated that GelMA@CINPs microspheres significantly enhanced NPC antioxidant capacity and promoted ECM secretion. Implantation of these microspheres into intervertebral discs (IVDs) of rats following discectomy validated their therapeutic efficacy in promoting NP tissue regeneration. In this experiment, the introduction of CINPs facilitates a dual antioxidant mechanism, comprising chemical (e.g., free radical scavenging by eumelanin via HAT/SET mechanisms) and biological (activation of the NRF2/HO-1 pathway) components. This synergistic approach directly addresses oxidative stress, a critical driver of intervertebral disc degeneration (IVDD) progression. This research introduces a novel strategy for improving cell-material interactions in tissue engineering, which enhances the potential for constructing an artificial NP and effectively treating IVDD.

## Graphical abstract

**Figure:**
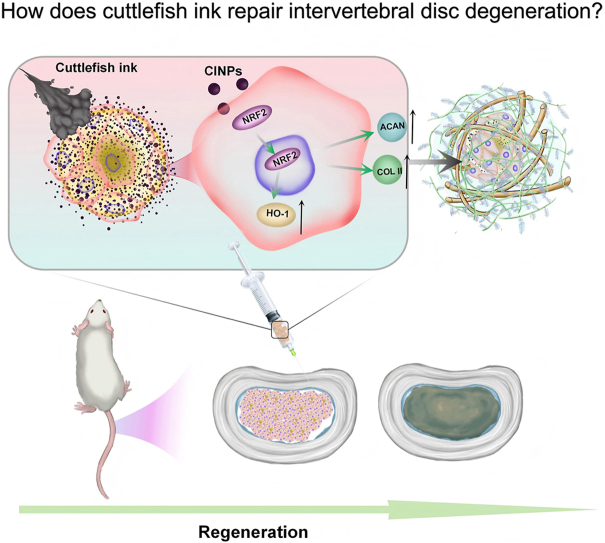

## 1 Introduction

The intervertebral disc (IVD), a specialized tissue located between adjacent vertebral bodies, comprises three main components: the central gelatinous nucleus pulposus (NP), the fibrous annulus fibrosus (AF), and the cartilaginous endplates [1]. These components function synergistically to absorb axial compressive forces, maintain spinal flexibility, and ensure biomechanical stability [2]. Intervertebral disc degeneration (IVDD), characterized by disrupted matrix metabolism and chronic inflammation, is a leading cause of chronic low back pain (LBP) [3]. This degeneration leads to disc height reduction, AF fissures, and altered biomechanical properties, causing pain and disability [4]. Initial treatments for IVDD include conservative approaches such as bed rest, physical therapy, and pharmacotherapy. As the condition progresses, surgical interventions like discectomy and spinal fusion may be necessary to alleviate pain and restore stability [5]. However, both conservative and surgical treatments have significant limitations. Conservative methods only provide temporary relief but do not halt degeneration, while surgeries can cause complications like adjacent segment degeneration and implant failure. Neither approach fully restores the disc's original structure and function or addresses the underlying degenerative processes [6]. Taken together, while current treatments offer some symptomatic relief and functional restoration, they fall short in providing long-term solutions for IVDD.

NP is a crucial component composed primarily of water, proteoglycans, and type II collagen fibers [7]. However, under conditions such as aging, mechanical stress, or injury, the ECM undergoes structural changes that lead to degeneration [8]. This degenerative process involves a vicious cycle where matrix breakdown products, such as pro-inflammatory cytokines and reactive oxygen species (ROS), accumulate due to impaired nutrient diffusion and oxygenation within the disc [9,10]. The vicious cycle of NP degeneration perpetuates itself as ROS-induced oxidative stress further damages cellular components, including proteoglycans and collagen fibers, accelerating matrix breakdown and altering the microenvironment of the disc [11]. Consequently, the NP loses its water content, becomes more fibrous, and loses its ability to effectively absorb mechanical loads. Due to the limited self-repair capacity of NP, regenerative medicine strategies, especially cell therapy [12,13], has emerged as promising approaches for treating IVDD. Cell therapy aims to replenish lost or dysfunctional NPCs and stimulate endogenous repair mechanisms. A variety of cell sources—mainly including NPCs and mesenchymal stem cells—have been evaluated in clinical trials [13]. Among them, combining NPCs with a three-dimensional scaffold represents a prominent form of artificial NP. This design simultaneously supplies viable cells and an instructive microenvironment that can be tuned for sustained delivery of bioactive cues [14,15]. Antioxidants also play a pivotal role in mitigating NP degeneration by neutralizing ROS and protecting the artificial NP from oxidative damage [12,16]. Enhancing antioxidant defenses through local administration may offer promising strategies to combat the detrimental effects of oxidative stress on the IVD in the cell therapy.

Cuttlefish ink has a long history of promoting human health in both Western cultures (especially ancient Greece and Rome) and Eastern cultures (such as China) [17]. Recently, it has regained attention in the development of new drugs due to its natural properties, health benefits, and cost-effectiveness. For example, Chen et al. revealed that the polysaccharides in Cuttlefish ink exert antioxidant effects by inhibiting the expression of Nicotinamide Adenine Dinucleotide Phosphate (NADPH) oxidase and connexins. Cuttlefish ink nanoparticles (CINPs), the main component of Cuttlefish ink, exhibit spherical shape and good dispersibility. Meanwhile, compared to other nanoparticles such as inorganic ones and nanofibers, CINPs exhibit natural biocompatibility and high repeatability [18]. They are rich in melanin, various amino acids, and monosaccharides, and have shown significant effects in antitumor [19], antibacterial [20], anti-inflammatory and antioxidant aspects. Eumelanin, the predominant melanin type in Cuttlefish ink, is renowned for its antioxidant properties. The chemical structure of eumelanin enables it to scavenge free radicals through two main mechanisms: hydrogen atom transfer (HAT) and single electron transfer (SET) mechanisms [21]. In HAT, eumelanin donates hydrogen atoms to neutralize ROS, effectively preventing oxidative damage to cellular structures. Conversely, in SET, eumelanin transfers single electrons to free radicals, stabilizing them and inhibiting chain reactions that lead to oxidative stress. The biological antioxidant activity of Cuttlefish ink extends beyond its direct antioxidant properties to involve the heme oxygenase-1 (HO-1) pathway. HO-1 is an enzyme that catalyzes the breakdown of heme into biliverdin, carbon monoxide (CO), and iron. These byproducts exhibit excellent antioxidant and anti-inflammatory effects within cells, contributing to cellular defense against oxidative stress [22].

Hydrogels are three-dimensional (3D) cross-linked network structures composed of hydrophilic polymers, characterized by high water content and porosity, thereby their physical properties are similar as those of the ECM [23]. Gelatin, a natural hydrogel derived from the hydrolysis of collagen [24], exhibits low immunogenicity [25] and contains various bioactive sequences, such as the arginine-glycine-aspartic acid (RGD) sequence, which can promote the adhesion and proliferation of multiple cell types [26]. The preparation of GelMA hydrogel typically involves reacting methacrylic anhydride with gelatin, followed by photo-crosslinking in the presence of a photoinitiator [27]. Utilizing microfluidic technology, GelMA hydrogel can be processed into microspheres with diameters of several hundred micrometers. Compared with traditional bulk hydrogels, these microspheres have a higher surface area-to-volume ratio, which enhances the cell-matrix interaction and promotes the rapid proliferation of cells on the surface of microspheres [28]. In addition, GelMA microspheres exhibit excellent injectability and drug sustained-release properties. Therefore, they hold broad application prospects as cell scaffold materials in tissue engineering.

**Scheme 1:**
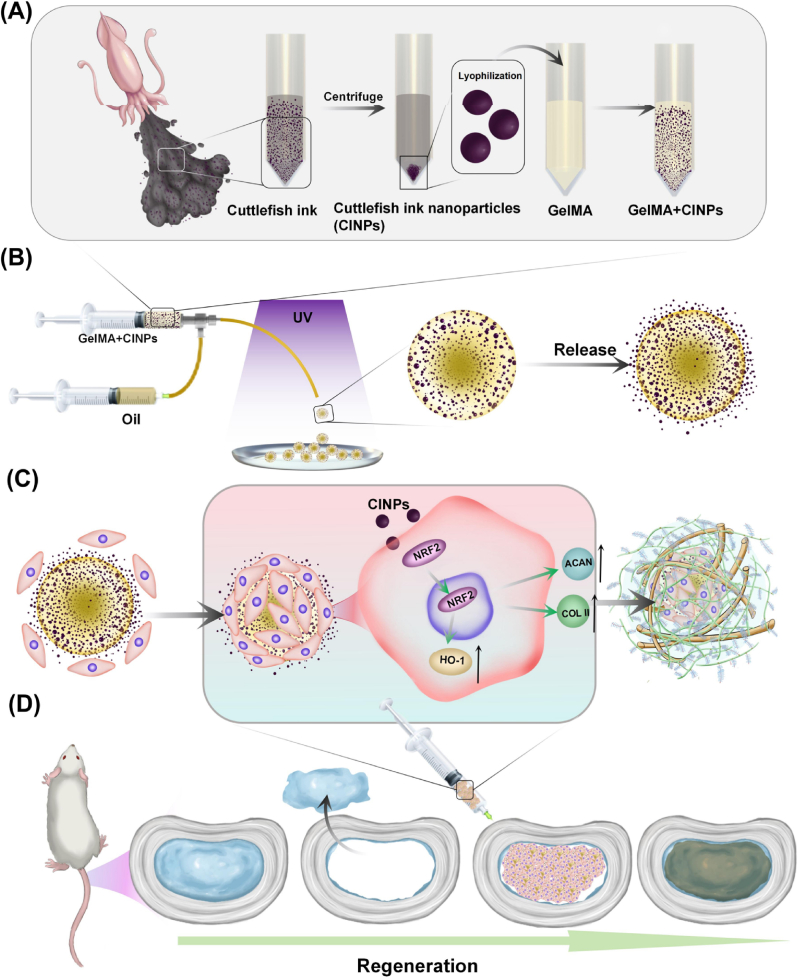
Schematic representation of NP tissue regeneration by cell-laden GelMA@CINPs. A, B) Separation of CINPs and preparation of GelMA@CINPs. C) The mechanism of antioxidant effects and NPCs regeneration promotion by GelMA@CINPs. D)The process of NP regeneration through the injection of cell-laden GelMA@CINPs.

As illustrated in Scheme 1, we will first conduct comprehensive characterization of CINPs, including component analysis, to gain an understanding of its potential implications. Then, we will develop GelMA@CINPs microspheres, which are composed of CINPs and GelMA hydrogel, utilizing microfluidic technology. It is worth emphasizing that our microspheres may exhibited excellent biocompatibility. Therefore, to investigate whether the GelMA@CINPs microspheres can protect NPCs and enhance their antioxidant capacity, we will subsequently load NPCs onto these microspheres to construct an artificial NP. The effects of GelMA@CINPs microspheres on matrix synthesis and antioxidant properties of NPCs will be proved. Mechanistically, we will perform transcriptome sequencing analysis. We will also explore the role of GelMA@CINPs microspheres on NPCs under oxidative stress conditions to further elucidate the underlying mechanisms. Finally, we will evaluate the tissue repair capability of our artificial NP by constructing a rat model of NP excision and injecting GelMA@CINPs microspheres loaded with NPCs into the IVD.

## 2 Materials and methods

### 2.1 Preparation and characterization of GelMA@CINPs hydrogel microspheres

#### 2.1.1 Synthesis of GelMA

The gelatin (Macleans, Shanghai, China) weighing 30 g was thoroughly mixed with 300 mL of phosphate-buffered saline (PBS) at a temperature of 60 °C. Subsequently, 16 mL of methacrylic anhydride (Aladdin, Shanghai, China) was slowly added to the solution using a microinjection pump at a rate of 0.25 mL/min under protected from light conditions. After a duration of 2 h, an aliquot of 800 mL of PBS was introduced to stop the reaction. The solution then underwent dialysis in distilled water using a dialysis bag at a temperature of 38 °C for 7 days. It was subsequently frozen at − 20 °C for 8 h and freeze-dried for 48 h. GelMA was successfully synthesized and confirmed by NMR with a grafting rate of 34.4 % (Fig. S1).

#### 2.1.2 Isolation of CINPs

Cuttlefish ink nanoparticles were extracted using the differential centrifugation method. Firstly, the Cuttlefish was dissected and the fresh ink sacs were eliminated. Then, a centrifuge was used at a speed of 2000 revolutions per minute (rpm) for 5 min to eliminate large particle impurities. Next, the supernatant was gathered and subjected to centrifugation at a speed of 12000 rpm for 10 min at 4 °C to obtain CINPs. Subsequently, the precipitate was washed three times with deionized water (DI water) and finally freeze-dried for three days using a freeze dryer to obtain CINPs.

#### 2.1.3 Preparation of GelMA@CINPs microspheres

First, completely dissolve 100 mg of GelMA in 1 mL of PBS solution containing 0.1 % photoinitiator lithium phenyl phosphinate (Sigma-Aldrich, St. Louis, MO, USA) at a temperature of 37 °C. Subsequently, uniformly mix 100 μg of CINPs into the GelMA solution and oscillate gently to ensure thorough combination. Employ a microfluidic device for the preparation of hydrogel microspheres comprising two microfluidic pumps and corresponding tubing with nozzles. The outer phase nozzle should have a diameter of 31G while the inner oil phase should consist of 5 % span 80 and mineral oil; the inner phase nozzle diameter should be set at 21G and contain GelMA@CINPs solution. Maintain an outer phase flow rate of 1 mL/min and an inward flow rate of 8 μL/min. Following irradiation with UV light at a wave length of 365 nm for 30 min, remove the oil phase and rinse the obtained microspheres using a mixture of 75 % ethanol and isopropanol (Sigma-Aldrich). Finally, subject them to freeze treatment for 48 h.

#### 2.1.4 Amino acid analysis

Take a 50 mg sample of CINPs and transfer it into a hydrolysis tube. Gradually add 4 mL of concentrated analytical grade hydrochloric acid (6 mol/L) in a 1:1 ratio. Subsequently, employ a nitrogen blower to blow nitrogen gas for 15 min to ensure thorough mixing before sealing the tube tightly. Then, place the sealed sample in an oven and conduct hydrolysis at 110 °C for 22–24 h. Once completed, remove the sample and allow it to cool down to room temperature prior to opening the tube. Adjust the sample solution volume up to a total of 100 mL, then precisely pipette out 2 mL for deacidification using a nitrogen blower at a temperature of 60 °C until completely dry. Afterward, thoroughly mix by adding hydrochloric acid with concentration of 0.02 mol/L on a vortex mixer. Finally, after filtration through a column with pore size of 0.22 μm, transfer the solution into an amino acid analyzer (MembraPure A300) for further analysis.

#### 2.1.5 Monosaccharide analysis

The CINPs (10 mg) were subjected to hydrolysis with 2 mL of trifluoroacetic acid (TFA) at 110 °C for 12 h under nitrogen protection. Unreacted TFA was subsequently eliminated at 50 °C using a gas blowing concentrator, followed by neutralization with 0.3 mol/L NaOH and dilution with water to reach a final volume of 1 mL. Ten monosaccharide standards, including L-arabinose, xylose, L-fucose, L-galactose, D-mannose, glucose, D-galacturonic acid, glucosamine, D-glucuronic acid and galactosamine were prepared as solutions with a concentration of 2 mmol/L each. Afterward, each solution (50 μL) was mixed with NaOH (0.3 mol/L; 450 μL) and PMP derivatization reagent (0.5 mol/L; 450 μL), heated in a water bath for 30 min and then cooled to room temperature. The reaction mixture was neutralized using HCl (0.3 mol/L; 450 μL) and extracted three times with chloroform (1 mL). Finally, the filtrate (20 μL) was passed through a filter with a pore size of 0.45 μm for high performance liquid chromatography analysis (Agilent 1260, USA).

#### 2.1.6 Characterization methods

The JEOL-2100 scanning transmission electron microscope (TEM) from the Japanese company JEOL was used to obtain photographs of CINPs. The CINPs were dispersed in water and subjected to 15 min of ultrasonic treatment before being measured for diameter using the Nanotrac Flex nanoparticle analyzer. Subsequently, their Zeta potential was measured using the Malvern Zetasizer Nano ZS90 instrument. The diameter of GelMA@CINPs microspheres was measured using the optical microscope (Zeiss Axiovert 200) from Carl Zeiss in Oberkochen, Germany. Scanning electron microscopy images of GelMA@CINPs microspheres were obtained using the Sigma scanning electron microscope from Zeiss in Germany. The chemical functional groups of various microspheres were characterized by Fourier transform infrared spectroscopy (FTIR, Thermo Scientific, USA) in the range of 4000–500 cm^−1^. Dried microspheres samples were ground into a fine powder, and their diffraction patterns were recorded. The chemical composition and valence states of the microspheres components were determined by an X-ray photoelectron spectroscopy (XPS, Thermo Scientific K-Alpha, USA). X-ray diffraction (XRD, SmartLab SE, Japan) was performed to evaluate the crystalline structure of the samples. By scanning over a range of 2θ angles and plotting the corresponding peak intensities, the crystalline characteristics of the samples were identified.

#### 2.1.7 Biodegradation assay of microspheres

The GelMA and GelMA@FD microspheres (100 mg) were individually immersed in PBS containing 0.25 mg/mL collagenase II (Sigma-Aldrich), and incubated on a shaker at 37 °C, 120 rpm with solution replacement every two days. Optical microscopy was employed to observe the microspheres throughout the first, second, third, and fourth weeks, while their weights were measured after freeze-drying.

### 2.2 In vitro evaluation of GelMA@CINPs microspheres on NPCs

#### 2.2.1 Isolation and culture of rat primary NPCs

The NP tissue was extracted from the IVDs of six-week-old SD rats, which were then euthanized. Subsequently, the tissue was placed in a culture dish and washed four times with PBS containing 10 % penicillin and streptomycin solution. Enzymatic digestion using 0.2 % collagenase type II at 37 °C for 1 h with gentle shaking was performed. After digestion, the cells were washed with PBS and transferred to a 75 cm^2^ culture flask in DMEM/F12 medium containing 10 % fetal bovine serum and 100 U/mL penicillin as well as 100 μg/mL streptomycin (all from Thermo Fisher Scientific), under a CO_2_ concentration of 5 % at 37 °C. Fresh medium was replaced every two days until cell growth reached 70 %–80 %, after which cell passage was conducted. Cells from passages P1-P3 would be utilized in subsequent experiments.

#### 2.2.2 NPCs culture on GelMA@CINPs microspheres

Sterilize the microspheres by immersing them in 75 % alcohol for 30 min, followed by three washes with PBS and air-drying in a six-well plate. Subsequently, add 100 μL of culture medium containing 500,000 cells and incubate at 37 °C for 30 min before supplementing with 2.5 mL of culture medium. Maintain the culture under conditions of 37 °C and 5 % CO_2_, changing the medium every two days.

#### 2.2.3 Cell viability and proliferation on GelMA@CINPs microspheres

In order to assess the impact of microspheres on cell proliferation, the carrier cells were cultured for 1, 3, and 5 days respectively. Subsequently, they were incubated in a culture medium containing a 10 % solution of cell counting kit-8 (CCK-8) for 1 h, and the absorbance was measured at a wavelength of 450 nm using a PowerWave XS spectrophotometer (BioTek, Winooski, VT, USA). To evaluate the viability of cells on microspheres, live/dead staining reagent was applied after three days of cultivation and incubated at 37 °C in darkness for 30 min. Finally, observation was conducted using a Zeiss Axiovert 40CFL fluorescence microscope (Carl Zeiss Inc.).

#### 2.2.4 Immunofluorescence staining

The cellular microspheres containing NPCs were initially stabilized by immersing them in a 4 % paraformaldehyde solution (Sigma Aldrich). This step was followed by a 10-min permeabilization step using 0.3 % Triton X-100 detergent (Sigma Aldrich). After a 20-min blocking process with a 5 % bovine serum albumin (BSA) solution, the samples were incubated overnight at 4 °C with primary antibodies specific for type II collagen (1:200, Abcam, Boston, MA, USA). Following thorough rinsing with PBS, the samples were treated with a secondary antibody, Alexa Fluor®488-conjugated goat anti-mouse IgG (1:1000, Abcam).Cytoskeletal structures were visualized through a 20-min staining with phalloidin-iFluor 594 (Abcam).The final step involved mounting the samples with Fluoroshield mounting medium containing DAPI nuclear counterstain(Abcam). Fluorescence imaging was conducted using specialized equipment from Carl Zeiss AG.

#### 2.2.5 Reverse transcription-quantitative polymerase chain reaction (RT-qPCR) assay

According to the protocol, TRIzol reagent (Thermo Fisher Scientific) was utilized for total RNA extraction from cells on microspheres. The cDNA synthesis was performed using a RevertAid First Strand cDNA Synthesis Kit (Thermo Fisher Scientific). Real-time PCR analysis was conducted employing the iTapTM Universal SYBR Green Supermix kit and the CFX96TM Real-Time PCR System (both from BioRad, Hercules, CA, USA). The expression levels of ECM-related genes, antioxidant enzymes, and mitochondrial respiratory chain factors were determined utilizing the comparative Ct (2 Ct) method with *Gapdh* (glyceraldehyde-3-phosphate dehydrogenase) as an internal reference. The primer sequences were designed by GenePharma (Shanghai, China).

#### 2.2.6 Western blot assay

The cell microspheres were incubated in RIPA lysis buffer (Beyotime) supplemented with a protease inhibitor cocktail (Thermo Fisher Scientific). Following centrifugation, the supernatant was collected and the protein concentration was determined using the BCA Protein Assay Kit (Beyotime). Loading buffer at 25 % concentration was added. Equal amounts of protein lysate were separated by 10 % sodium dodecyl sulfate-polyarylamide gel electrophoresis (SDS-PAGE) for 1.5 h at 100V, then transferred to polyvinylidene difluoride (PVDF) membranes for 1 h at a constant current of 350 mA. The blotted membranes were blocked with a blocking buffer (Beyotime) for 30 min and subsequently incubated overnight with primary antibodies. After washing three times with TBST, goat anti-mouse or anti-rabbit horseradish peroxidase-conjugated secondary antibodies (Abcam) were applied. Chemiluminescence reagent from Millipore, Billerica, MA, USA was used to visualize the results on a Bio-Rad ChemiDoc Touch Imaging System. ImageJ software was utilized for protein concentration analysis and β-actin bands served as standards for result normalization.

#### 2.2.7 Oxidative stress intervention

The NPCs and microspheres were co-cultivated in a standard complete medium at a temperature of 37 °C under the presence of 5 % CO_2_ for a duration of 48 h. Subsequently, the culture was subjected to an intervention by replacing the complete medium with one containing hydrogen peroxide (H_2_O_2_) at a concentration of 1 mM, which lasted for up to 24 h under similar conditions.

#### 2.2.8 Intracellular ROS staining

After oxidative stress intervention, the cells were washed with PBS and incubated with DCFH-DA solution (10 μM) at 37 °C for 20 min. After washing with PBS, the samples were observed using a fluorescence microscope (Carl Zeiss Inc.).

#### 2.2.9 Small interfering RNA (siRNA) transfection

To achieve transient silencing of Nuclear Factor erythroid 2-Related Factor 2 (NRF2) in NPCs, siRNA targeting *Nrf2* was obtained from GenePharma. NPCs were transfected with either 100 nM *Nrf2*-specific or negative control (NC) siRNA using Lipofectamine 2000 (Thermo Fisher Scientific), following the protocol provided by the manufacturer. Specifically, 1 × 10^6^ cells were seeded in each well of a 6-well plate and incubated with Opti-MEM medium (Thermo Fisher Scientific) containing the corresponding siRNA for 24 h before transfection. After 8 h of siRNA treatment, the medium was replaced with fresh growth media. Subsequently, NPCs were harvested and transferred onto GelMA@CINPs microspheres for further experiments. The sequences used for *Nrf2* siRNA were: Sense (5′–3′): GGGUAAGUCGAGAAGUGUUTT; Antisense (5′–3′): AACACUUCUCGACUUACCCTTGCGCGC.

### 2.3 Transcriptomic analysis

The NPCs cultured on either GelMA or GelMA@CINPs microspheres were subjected to total RNA isolation using the TRIzol reagent, following the manufacturer's protocol. Subsequently, the RNA concentration was quantified using a NanoDrop 2000 spectrophotometer (Thermo Fisher Scientific), and the integrity of the RNA was assessed with an Agilent 2100 Bioanalyzer (Agilent Technologies, Santa Clara, CA, USA). Library preparation was performed according to provided guidelines utilizing the TruSeq Stranded mRNA LT Sample Prep Kit (Illumina, San Diego, CA, USA). Next-generation sequencing (NGS) was then conducted on the Illumina HiSeq X Ten platform by OE Biotechnology Co. (Shanghai, China) to obtain more precise gene expression profiles for each sample. The mapped reads were obtained by aligning them to reference genomes using Trimmomatic software coupled with HISAT2. To identify enriched biological processes and pathways, Gene Ontology (GO) and Kyoto Encyclopedia of Genes and Genomes (KEGG) enrichment analyses were carried out employing DAVID. Genes were considered significantly differentially expressed if they exhibited a p-value <0.05 and FC > 1.5 or <0.5.

### 2.4 In vivo experiments to reconstruct rat lumbar NP tissue

#### 2.4.1 Ethics statement

The Ethics Committee of Soochow University approved all animal experiments (Approval No. SUDA20240611A05). Sixty six-week-old male SD rats (180 ± 15 g) were purchased from the Animal Center of Soochow University and housed in an environment with constant humidity (50–60 %), temperature (22–24 °C), and a light cycle from 6 a.m. to 6 p.m.

#### 2.4.2 Surgical induction of a rat tail NP discectomy model

SD rats were anesthetized with intraperitoneal injection of 3 % Sodium Pentobarbital (1.5 mL/kg body weight; Shanghai Merck Co., Ltd., China). A longitudinal incision was made 2 cm from the anus on the tail vertebrae, and after separating the ligament, a 22-gauge needle was inserted into the fibrous ring to aspirate NP tissue using a 5 mL syringe. The remaining NP was removed using a curette. The rats were divided into seven groups: Sham, Discectomy, NPCs, GelMA, GelMA + NPCs, GelMA@CINPs and GelMA@CINPs + NPCs group. After removal of the NP, either a cell suspension or microsphere solution (10 μL) was injected using a syringe and then the fibrous ring was sutured. Finally, penicillin (80,000 Units/rat) was injected into the thigh muscle for three days.

#### 2.4.3 Diagnostic imaging

After 4 and 8 weeks postoperatively, rats were anesthetized and underwent X-ray imaging (RAD speed M, Shimadzu, Japan) with the following parameters: 250 mA, 50 kV, and 20 msec. The surgical outcomes were evaluated using the DHI index. To assess the hydration level of NP, rats underwent T2-weighted sagittal MRI scans (GE Signa HDe 1.5 T Equipment, General Electric Company, Boston, MA, USA) with the following settings: TR = 3500 ms, TE = 102 ms and section thickness = 1.4 mm. Images were measured using Image J software and then graded according to Piffirmann classification by two independent individuals (J.C.Y. and D.J.Q.) in a blinded manner.

#### 2.4.4 Histology

The rats were euthanized at 4 and 8 weeks, and the IVDs containing the modeling were dissected. The specimens were then fixed in 4 % paraformaldehyde for one week, followed by immersion in a solution of 10 % ethylenedinitrilotetraacetic acid (EDTA; SigmaAldrich) for a duration of 6 weeks. After removal, the samples were embedded in paraffin. Subsequently, thin slices with a thickness of 5 mm were obtained from the paraffin-embedded samples using a Leica microtome (Leica Biosystems, Buffalo Grove, IL, USA). The sections were then deparaffinized and subjected to staining with hematoxylin and eosin (HE), as well as Safranin O-fast green (S.O.), to visualize the structure of IVDs and protein polysaccharide deposition. Immunohistochemistry was employed to assess the expression level of Collagen II in the NP. Finally, observations were made using a Zeiss Axiovert 40CFL microscope. Two independent observers (J.C.Y. and D.J.Q.) jointly conducted histological scoring based on a pre-established grading system.

#### 2.4.5 Statistical analysis

All data were presented as mean ± standard deviation (S.D.). Statistical analysis was conducted using SPSS 14.0 software (SPSS Inc., Chicago, USA). Comparisons between the two groups were performed using Student's t-test, while comparisons among multiple groups were conducted using one-way Analysis of Variance (ANOVA) followed by Tukey's post hoc test. P values less than 0.05 (∗) or less than 0.01 (∗∗) were considered statistically significant.

## 3 Results

### 3.1 Characterization of CINPs and microspheres

**Fig. 1:**
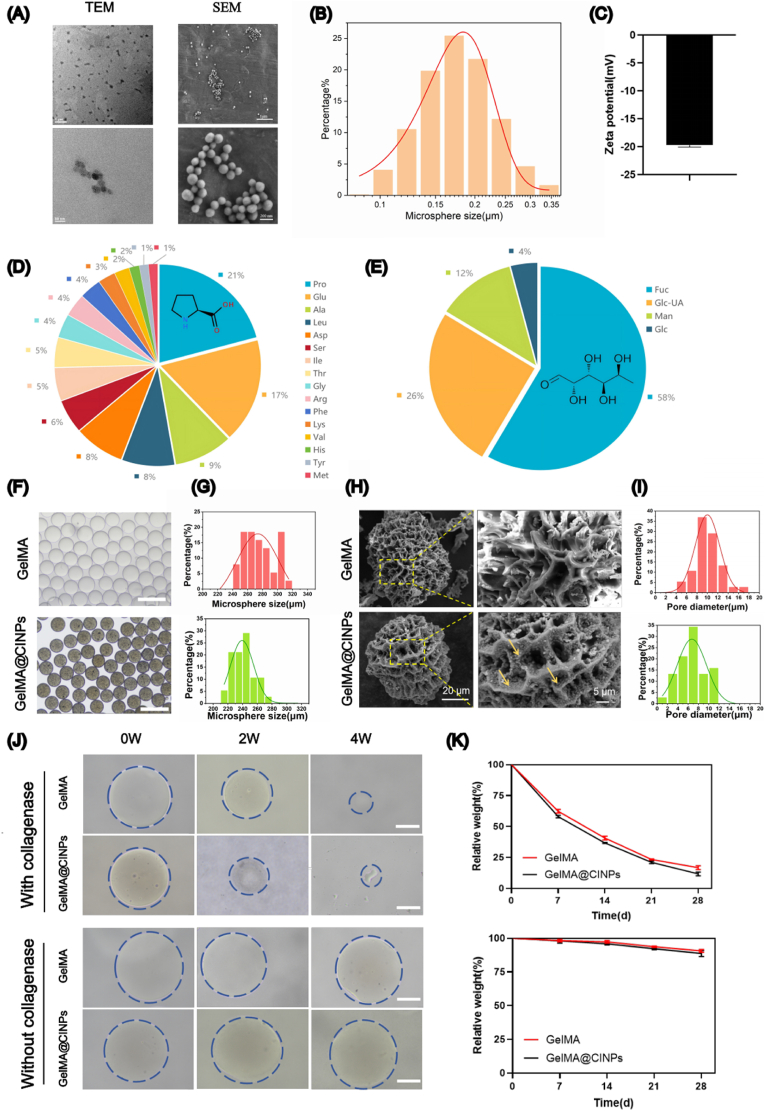
Characterizations of CINPs and GelMA@CINPs. A) TEM images of CINPs and SEM images of CINPs. B) Hydrodynamic size distribution of CINPs. C) Zeta potential of CINPs. D) Amino acid analysis of CINPs. E) Monosaccharide analysis of CINPs. F) Optical microscope images of GelMA and GelMA@CINPs. Scale bar = 500 μm. G) The size distribution of GelMA and GelMA@CINPs. H) SEM images of GelMA and GelMA@CINPs. I) Pore size distribution of GelMA and GelMA@CINPs. J) Optical microscope images of GelMA and GelMA@CINPs with or without collagenase treatment for 0, 2, and 4 weeks. Scale bar = 100 μm. K) Quantitative analysis of degradation assay. Data are presented as mean ± SD of three independent experiments (n = 3) for degradation assay.

Following extraction via differential centrifugation, CINPs were observed under SEM and TEM, revealing a spherical morphology with an approximate diameter of 100 nm (Fig. 1A). The hydrodynamic size of CINPs was approximately 166.8 nm (Fig. 1B), with a zeta potential of −19.7 mV (Fig. 1C), indicating a propensity for aggregation and resistance to clearance within tissues. Amino acid analysis showed that amino acids constituted 12.07 % of the mass of CINPs, including 21 % proline and 17 % glutamic acid (Fig. 1D). Monosaccharide analysis indicated that CINPs primarily consisted of fucose (58 %), with additional presence of glucuronic acid and mannose (Fig. 1E). The FTIR spectra of GelMA and GelMA@CINPs microspheres showed no significant differences, indicating that no new chemical bonds were formed between CINPs and GelMA (Fig. S2). The full XPS spectra revealed that both GelMA and GelMA@CINPs microspheres mainly contained carbon, nitrogen, and oxygen. Interestingly, the incorporation of CINPs did not cause any obvious changes in the XPS spectra of the microspheres, which may be attributed to the low content of CINPs being below the detection limit (Fig. S3A, Fig. S3B). The XRD spectra indicated that both GelMA and GelMA@CINPs microspheres are amorphous (Fig. S3C).

Following microscopic characterization and chemical analysis of CINPs, further testing was conducted on microspheres. Microscopic examination revealed that GelMA and GelMA@CINPs exhibited good dispersibility and integrity in water (Fig. 1F), appearing as uniform spheres with diameters around 300 μm (Fig. 1G). Presence of CINPs imparted a dark gray color to GelMA@CINPs microspheres. SEM analysis demonstrated that both GelMA and GelMA@CINPs microspheres possessed porous structures (Fig. 1H), with pore diameters of approximately 5 μm for GelMA microspheres and 10 μm for GelMA@CINPs microspheres. Additionally, particle-like substances were observed embedded on the surface of GelMA@CINPs microspheres, confirming successful loading of CINPs onto the microspheres (Fig. 1I).

In vitro degradation experiments indicated that both types of microspheres retained less than 20 % of their mass after 4 weeks of collagenase treatment. However, in the absence of collagenase, neither of the two types of microspheres degraded significantly (Fig. 1J and K). Culturing of NPCs on these microspheres for five days revealed effective cell adhesion and proliferation, with similar growth states observed on both materials, demonstrating good adhesion and extension capabilities.

### 3.2 In vitro cytocompatibility of GelMA@CINPs microspheres

Optimal cell compatibility is crucial for the regeneration of NPCs on microspheres. Therefore, we evaluated the cell morphology, viability and proliferation of NPCs cultured on these microspheres.

**Fig. 2:**
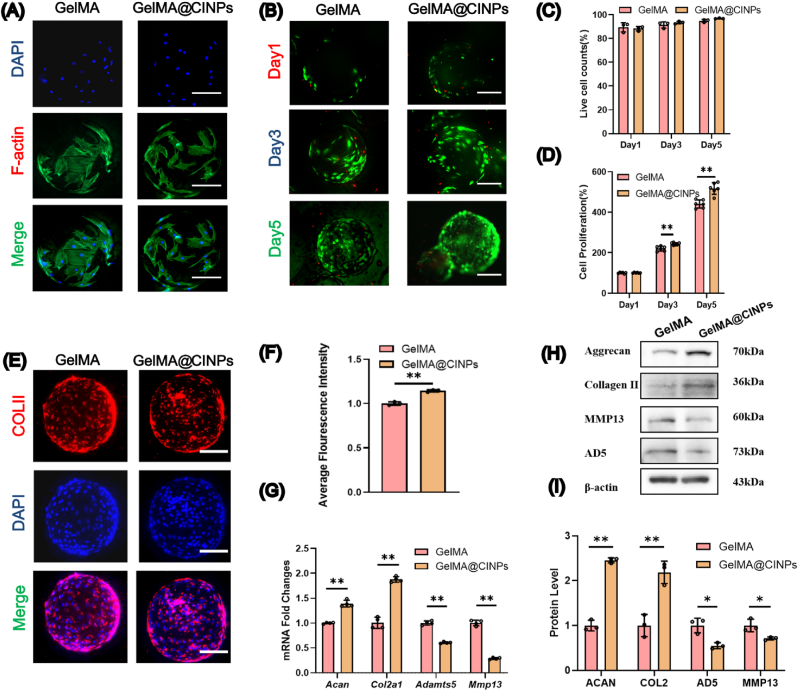
In vitro cytocompatibility of GelMA@CINPs and the effect on matrix synthesis of NPCs. A) Immunofluorescence staining of the cytoskeleton of cells on microspheres. B) Live/Dead cell staining of NPCs cultured on GelMA or GelMA@CINPs at time points of days 1, 3, and 5. Scale bar = 100 μm. C) Quantification of live NPCs cultured on GelMA or GelMA@CINPs at time points of days 1, 3, and 5. D) CCK-8 assay of NPCs cultured on GelMA or GelMA@CINPs at time points of days 1, 3, and 5. E) Immunofluorescence staining of COL II in NPCs cultured on GelMA or GelMA@CINPs. F) Quantification of COL II in NPCs cultured on GelMA or GelMA@CINPs. G) The mRNA expressions of matrix synthesis and degradation genes, *Acan*, *Col2a1*, *Adamts5* and *Mmp1*3 by RT-qPCR in NPCs. H) Western blot analysis of NP matrix synthesis and degradation proteins in NPCs. I) The semi-quantitative analysis of Western blot analysis. Data are expressed as the mean ± SD derived from six independent experiments (n = 6) for CCK-8 assays, four independent experiments (n = 4) for RT-qPCR, and three independent experiments (n = 3) for Western blot analysis. Statistically significant differences are denoted by ∗ (p < 0.05) or ∗∗ (p < 0.01) when comparing the specified groups.

Culturing of NPCs on these microspheres for five days revealed effective cell adhesion and proliferation, with similar growth states observed on both materials, demonstrating good adhesion and extension capabilities (Fig. 2A). Live-dead cell staining indicated excellent cell adhesion on both types of microspheres, with minimal cell death observed on days 1, 3, and 5, resulting in a survival rate of approximately 90 % (Fig. 2B and C). CCK-8 assays demonstrated sustained proliferation of NPCs on the microspheres, with faster cell proliferation observed on GelMA@CINPs microspheres compared to GelMA microspheres, suggesting a potential cell-promoting effect of CINPs (Fig. 2D). We also compared the differences in cell growth between traditional culture plates and microspheres. The results showed that cells cultured on microspheres exhibited significantly upregulated expression of the *Acan* and *Col2a1* genes, indicating that GelMA microspheres enhance the matrix synthesis capacity of the cells. In contrast, the expression of *Adamts5* and *Mmp13* genes was significantly downregulated, suggesting that GelMA microspheres inhibit matrix degradation (Fig. S4). This demonstrates that GelMA microspheres with a three-dimensional structure are more suitable for cell culture. Therefore, in subsequent experiments, we chose GelMA microspheres as the carrier for further studies.

### 3.3 Effects of GelMA@CINPs microspheres on the matrix synthesis of NPCs

Collagen II immunofluorescence showed stronger brightness in the GelMA@CINPs group compared to the GelMA group, with quantitative analysis revealing an average fluorescence intensity 14 % higher in the GelMA@CINPs group (Fig. 2E and F). Furthermore, RT-qPCR indicated significant upregulation of ECM synthesis-related genes including *Acan* and *Col2a1*, increasing by 39 % and 88 % respectively, while matrix degradation genes *Adamts5* and *Mmp13* were significantly downregulated, decreasing by 71 % and 39 % respectively (Fig. 2G). Additionally, Western blot analysis further demonstrated that protein levels of ACAN and COLII increased by 145.3 % and 118.4 % respectively, whereas protein levels of ADAMTS5 and MMP13 decreased by 46.2 % and 28.9 % (Fig. 2H and I). These findings illustrate that CINPs can enhance matrix synthesis and reduce matrix degradation, thereby increasing the overall ECM content of the NP.

### 3.4 RNA sequencing analysis of NPCs harvested from hydrogel microspheres

**Fig. 3:**
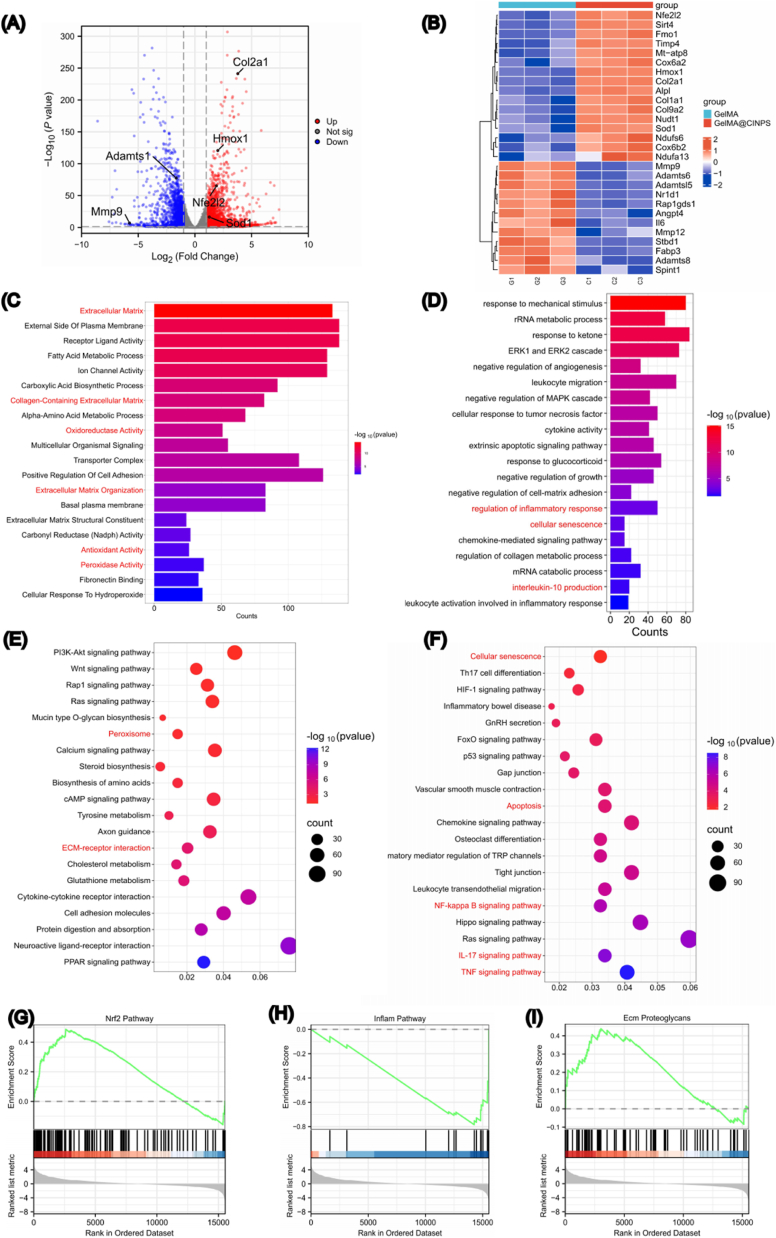
RNA sequencing analysis of NPCs harvested from hydrogel microspheres. A) Volcano plot for Differentially expressed genes.The red dots represent upregulated genes,and the blue dots represent downregulated genes. B) Heatmap for matrix and antioxidant-related genes. C) GO enrichment analysis of biological processes and cellular components, illustrating the number of up-regulated genes. D)GO enrichment analysis of biological processes and cellular components, illustrating the number of down-regulated genes. E) KEGG analysis of the up-regulated signaling pathways. F) KEGG analysis of the down-regulated signaling pathways. G,H,I)GSEA analysis demonstrated that NPCs cultured with GelMA@CINPs exhibited a positive correlation with the NRF2 Pathway and ECM proteoglycans, while showing a negative correlation with the Inflam Pathway. (For interpretation of the references to color in this figure legend, the reader is referred to the Web version of this article.)

To delve into the mechanisms of microspheres on cell function and the molecular mechanisms underlying their promotion of matrix synthesis in NPCs, this study employed RNA sequencing (RNA-seq) to detect differentially expressed genes between the GelMA group and the GelMA@CINPs group. The results showed that compared with the GelMA group, 2497 genes were upregulated and 1610 genes were downregulated in the GelMA@CINPs group. Volcano and heatmap analyses revealed that genes related to matrix synthesis, such as *Col2a1*, were significantly upregulated in the GelMA@CINPs group, while genes associated with antioxidant functions, such as *Nfe2l2* and *HMOX1*, were significantly increased, and genes related to matrix degradation, such as *Mmp9* and *Adamts1*, were significantly decreased (Fig. 3A and B). Further Gene Ontology (GO) analysis indicated that upregulated genes were mainly involved in ECM synthesis and antioxidant pathways (Fig. 3C), while downregulated genes were primarily associated with cellular senescence and inflammation-related pathways (Fig. 3D). Kyoto Encyclopedia of Genes and Genomes (KEGG) enrichment analysis showed that the Peroxisome and ECM-receptor interaction pathways were upregulated (Fig. 3E), while Cellular senescence, Apoptosis, NF-kappa B signaling pathway, IL-17 signaling pathway, and TNF signaling pathway were downregulated (Fig. 3F). Additionally, Gene Set Enrichment Analysis (GSEA) showed that the GelMA@CINPs group was positively correlated with the NRF2 Pathway and ECM proteoglycans, and negatively correlated with the inflammation pathway (Fig. 3G–H, Fig. 3I). The comprehensive analysis indicated that GelMA@CINPs could enhance the matrix synthesis and antioxidant capacity of NPCs, while reducing matrix degradation and inflammatory stress.

### 3.5 GelMA@CINPs microspheres enhanced the antioxidant function of NPCs

**Fig. 4:**
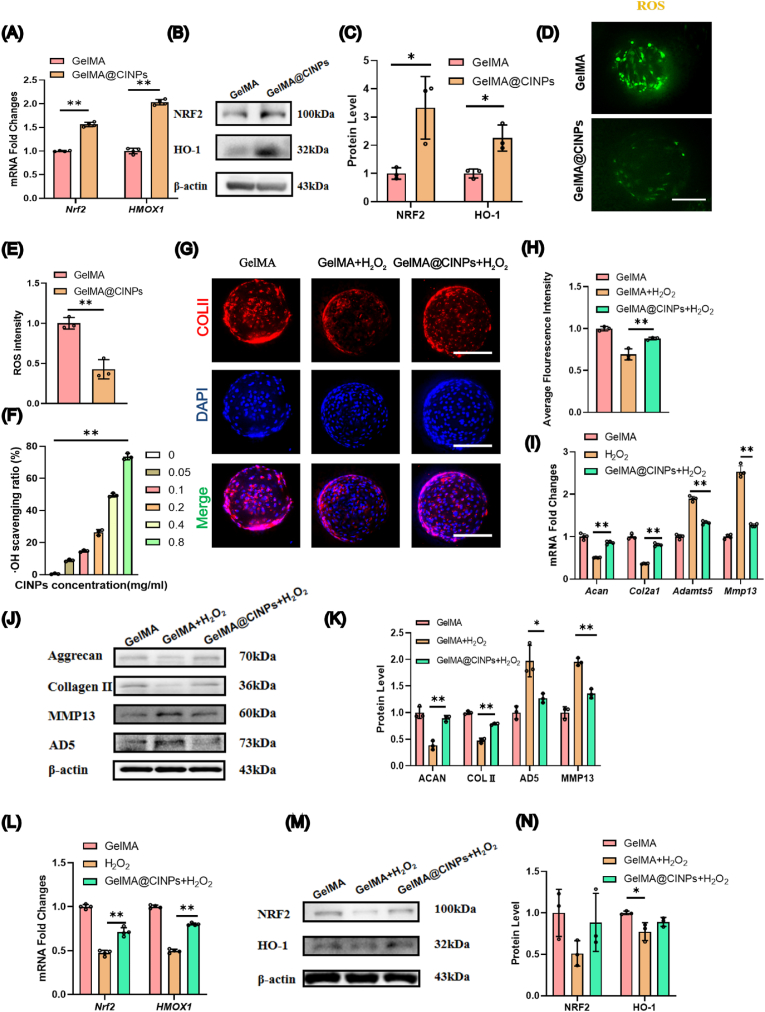
The antioxidant capacity and mechanism of GelMA@CINPs. A) The mRNA expressions of antioxidant-related genes, *Nrf2* and *HMOX1* by RT-qPCR in NPCs cultured on GelMA or GelMA@CINPs. B) Western blot analysis of NRF2 and HO-1 proteins in NPCs cultured on GelMA or GelMA@CINPs. C) The semi-quantitative analysis of Western blot analysis. D) The degree of intracellular reactive ROS in NPCs cultured with GelMA or GelMA@CINPs was visualized through DCFH-DA-based immunofluorescence staining. Scale bar = 100 μm. E) Quantification of DCFH-DA-based immunofluorescence staining. F) The scavenging rate of ·OH by CINPs at different concentrations. G) Immunofluorescence staining of COL II in NPCs cultured on GelMA, GelMA with H_2_O_2_, and GelMA@CINPs with H_2_O_2_. H) Quantification of COL II immunofluorescence staining. Scale bar = 150 μm. I) The mRNA expressions of matrix synthesis and degradation genes, *Acan*, *Col2a1*, *Adamts5* and *Mmp1*3 by RT-qPCR in NPCs cultured on GelMA, GelMA with H_2_O_2_, and GelMA@CINPs with H_2_O_2_. J) Western blot analysis of NP matrix synthesis and degradation proteins in NPCs cultured on GelMA, GelMA with H_2_O_2_, and GelMA@CINPs with H_2_O_2_. K) The semi-quantitative analysis of Western blot analysis. L) The mRNA expressions of antioxidant-related genes, *Nrf2* and *HMOX1* by RT-qPCR in NPCs cultured on GelMA, GelMA with H_2_O_2_, and GelMA@CINPs with H_2_O_2_. M) Western blot analysis of NRF2 and HO-1 proteins in NPCs cultured on GelMA, GelMA with H_2_O_2_, and GelMA@CINPs with H_2_O_2_. N) The semi-quantitative analysis of Western blot analysis. Data are expressed as the mean ± SD derived from four independent experiments (n = 4) for RT-qPCR, and three independent experiments (n = 3) for Western blot analysis, immunofluorescence staining and ·OH scavenging experiment. Statistically significant differences are denoted by ∗ (p < 0.05) or ∗∗ (p < 0.01) when comparing the specified groups.

To further validate the sequencing results, RT-qPCR indicated significant upregulation of antioxidant-related genes *Nrf2* and *heme oxygenase-1* (*HMOX1)* by 56 % and 104 % respectively (Fig. 4A). Western blot analysis showed notable increases in NRF2 protein and HO-1 protein by 233.2 % and 125.6 % respectively (Fig. 4B and C). Additionally, immunofluorescence revealed a significant decrease in DCFDA fluorescence (an indicator of total intracellular ROS) by 57.3 % in the GelMA@CINPs group compared to GelMA, suggesting activation of the NRF2/HO-1 antioxidant signaling pathway by CINPs (Fig. 4D and E). Interestingly, as the concentration increases, CINPs can significantly enhance the scavenging capacity of ·OH (Fig. 4F).

To confirm the antioxidant effects of CINPs, NPCs were subjected to oxidative stress. Under H_2_O_2_ intervention, Collagen II immunofluorescence showed reduced fluorescence intensity in the GelMA group, whereas the GelMA@CINPs group exhibited a slight decrease compared to the normal group. Quantitative analysis indicated a 30.9 % decrease in the GelMA group and a 12.9 % decrease in the GelMA@CINPs group (Fig. 4G and H). RT-qPCR revealed significant downregulation of matrix synthesis-related genes *Aggrecan* (49.3 % decrease) and *Col2a1* (63.8 % decrease) in the GelMA group under H_2_O_2_ intervention, with upregulation of matrix-degrading enzymes *Adamts5* (89.3 % increase) and *Mmp13* (153.0 % increase). Interestingly, in the GelMA@CINPs group, *Aggrecan* and *Col2a1* were downregulated by 13.9 % and 19.3 % respectively, while *Adamts5* and *Mmp13* were upregulated by 33.2 % and 27.2 % respectively (Fig. 4I).

Western blot analysis showed similar trends (Fig. 4J and K). This indicates that the balance between matrix synthesis and degradation is disrupted in the GelMA group under oxidative stress, whereas CINPs enhance the antioxidant capacity of NPCs, reducing external damage. To verify this hypothesis, exploration of the NRF2/HO-1 pathway was conducted. Under H_2_O_2_ intervention, RT-qPCR showed *Nrf2* decreased by 53 % and *HMOX1* by 50 % in the GelMA group, whereas in the GelMA@CINPs group, *Nrf2* decreased by 29 % and *HMOX1* by 20 % (Fig. 4L). Western blot analysis indicated NRF2 decreased by 48.9 % and HO-1 by 26.7 % in the GelMA group, and by 11.5 % and 10.9 % respectively in the GelMA@CINPs group (Fig. 4M and N). This suggests that the control group experienced severe disruption in antioxidant systems under oxidative stress beyond their regulatory threshold, while CINPs mitigated H_2_O_2_ damage, enhancing NRF2 and HO-1 expression to exert antioxidant effects.

### 3.6 NRF2 is required for antioxidant functions of GelMA@CINPs-cultured NPCs

In order to validate the transcriptomic sequencing results identifying the NRF2/HO-1 pathway, we performed targeted siRNA interference against *Nrf2* in NPCs, which were then seeded onto GelMA@CINPs microspheres.Transfection with *Nrf2*-targeted siRNA resulted in a significant reduction of NRF2 expression at both the mRNA (Fig. S5A) and protein levels (Fig. S5B, Fig. S5C), with the expression of NRF2 reduced to less than 30 % of that in the NC group, indicating the successful establishment of the *Nrf2*-knockdown nucleus pulposus cell model. Inhibition of *Nrf2* led to decreased expression of *HMOX1* mRNA expression (Fig. S5A) and HO-1 protein levels (Fig. S5B and C). Suppression of the NRF2/HO-1 pathway caused a marked increase in intracellular ROS (Fig. S5D, Fig. S4E), which is associated with diminished antioxidant capacity of NPCs. In addition, inhibition of *Nrf2* significantly reduced the matrix synthesis capacity of NPCs at both the mRNA (Fig. S5F) and protein levels (Fig. S5G, Fig. S5H), while markedly increasing the matrix degradation capacity at both the mRNA (Fig. S5F) and protein levels (Fig. S5G, Fig. S5H). Furthermore, Collagen II immunofluorescence showed weaker signal intensity in the *siNrf2* group compared to the NC group (Fig. S5I, Fig. S5J). Collectively, these results indicate that GelMA@CINPs microspheres effectively enhance the intracellular antioxidant capacity and matrix synthesis ability of NPCs by activating the NRF2/HO-1 signaling pathway.

### 3.7 Therapeutic efficacy of NPCs-laden GelMA@CINPs microsphere in vivo

**Fig. 5:**
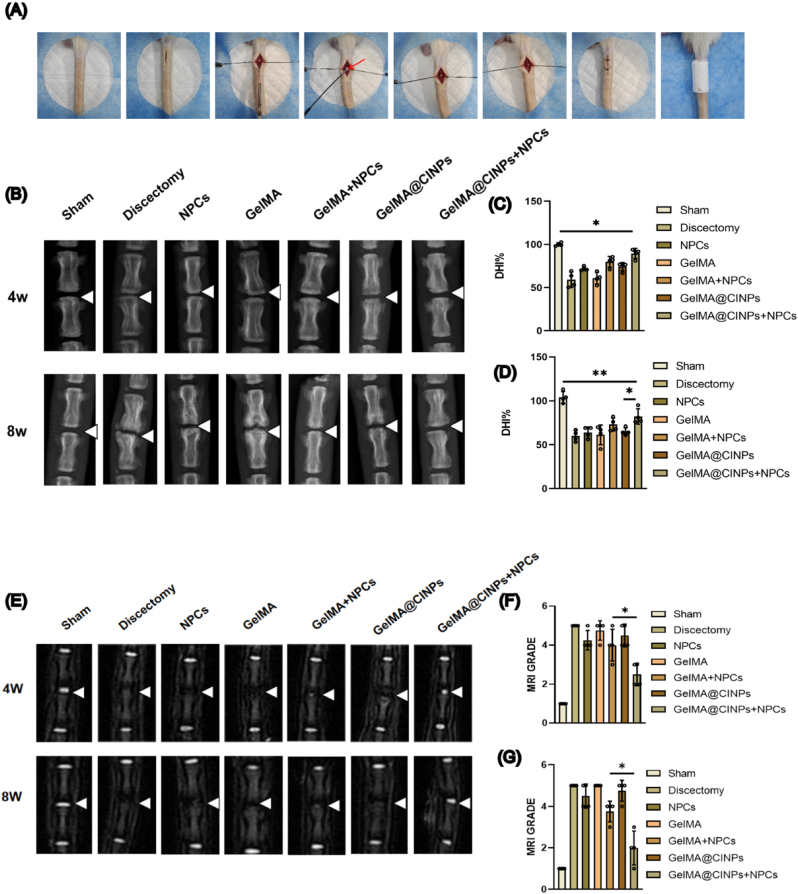
Radiological assessment of cell-laden GelMA@CINPs for NP regeneration in rat discectomized IVDs. A) The overall process of animal experiments. B) X-ray images of the Sham, Discectomy and treated IVDs at 4 and 8 weeks post-treatment. C,D) The disc height index (DHI) of the seven groups at 4 and 8 weeks post-treatment. E) T2 MRI images of the Sham, Discectomy and treated IVDs at 4 and 8 weeks post-treatment. F,G) Pfirrmann grading of IVD degeneration from the MRI images of the seven groups at 4 and 8 weeks post-treatment. Data are presented as mean ± SD of four independent experiments (n = 4) for Pfirrmann grading and DHI assessment.Statistically significant differences are denoted by ∗ (p < 0.05) or ∗∗ (p < 0.01) when comparing the specified groups.

To validate the effectiveness of artificial NP in vivo, we performed IVD surgery and injected NPCs into the rat tail vertebrae (Fig. 5A). The study included Sham, Discectomy, NPCs, GelMA, GelMA + NPCs, GelMA@CINPs, and GelMA@CINPs + NPCs groups. After 4 and 8 weeks of treatment, we conducted imaging evaluations using X-ray to assess changes in disc height and MRI to measure NP hydration. Changes in disc height, quantified using DHI%, showed that at 8 weeks, the Discectomy, GelMA, NPCs, and GelMA@CINPs groups decreased by about 40 % compared to the Sham group. The GelMA + NPCs group decreased by 27 %, whereas the GelMA@CINPs + NPCs group only decreased by 18 %, suggesting disc regeneration (Fig. 5B–C, Fig. 5D). MRI results showed that the Sham group exhibited white high-intensity signals indicative of high hydration levels in the natural NP. The Discectomy, GelMA, and GelMA@CINPs groups showed black high-intensity signals, indicating that the NP was removed and not regenerated. The NPCs group exhibited minimal white signals, suggesting that cell injection alone did not effectively form new NP tissue. The GelMA + NPCs group showed slight white high-intensity signals, indicating that GelMA provided a scaffold for new NP tissue formation. Interestingly, the GelMA@CINPs + NPCs group exhibited moderate white high-intensity signals, showing the best therapeutic effect among the treatment groups (Fig. 5E). Pfirrmann's grading system demonstrated that at 8 weeks, the Discectomy, GelMA, and GelMA@CINPs groups had approximately 4 times higher grades compared to the Sham group. The NPCs and GelMA + NPCs groups were 3.5 and 2.75 times higher respectively, while the GelMA@CINPs + NPCs group was only 1.4 times higher. The trends in treatment effects at 4 weeks and 8 weeks were consistent (Fig. 5F and G).

**Fig. 6:**
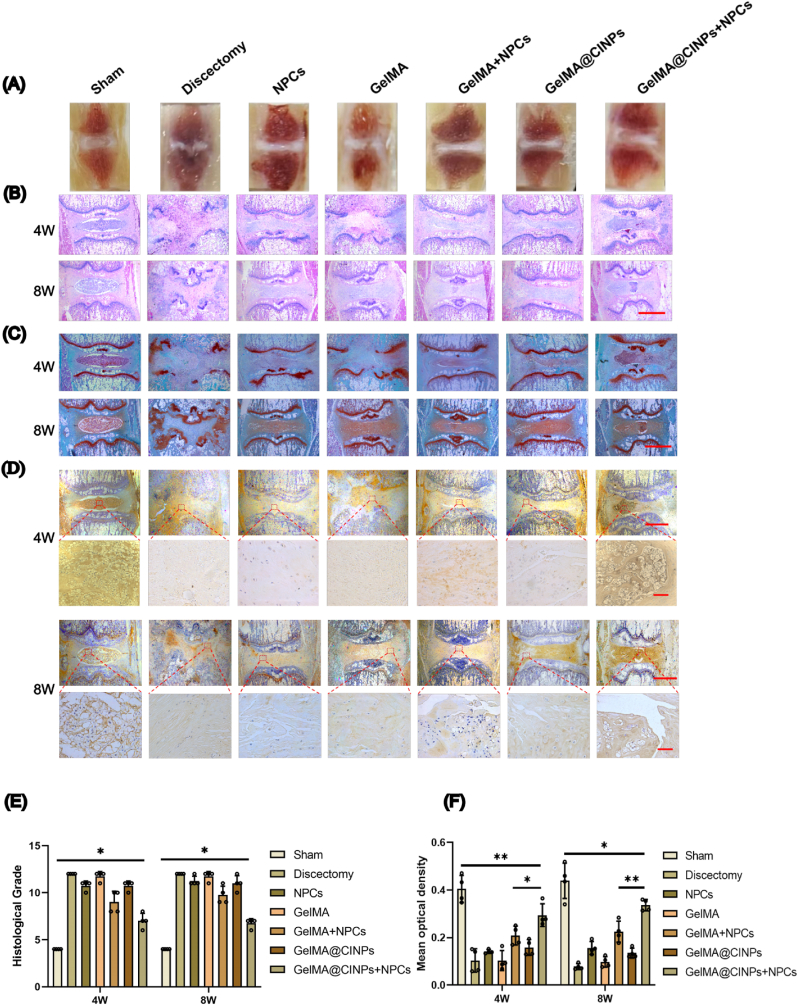
Histological evaluation of cell-laden GelMA@CINPs for NP regeneration in rat discectomized IVDs. A) Representative images of gross pathology of NP tissues in IVDs post-treatment. B) Representative images of hematoxylin and eosin (H&E) staining at 4 and 8 weeks post-treatment. Scale bar = 1.5 mm. C) Representative images of Safranin O (S.O) staining at 4 and 8 weeks post-treatment. Scale bar = 1.5 mm. D) Representative images of IHC staining for type II collagen (collagen II) at 4 and 8 weeks post-treatment. Scale bar = 1.5 mm (upper panels). Scale bar = 40 μm (lower panels). E) Histological scores for IVDs in each group at 4 and 8 weeks post-treatment. F) The quantitative analysis of COL-II of different groups at 4 and 8 weeks post-treatment. Data are presented as mean ± SD of four independent experiments (n = 4) for histological scoring assessment and quantitative analysis of IHC staining. Statistically significant differences are denoted by ∗ (p < 0.05) or ∗∗ (p < 0.01) when comparing the specified groups.

Further evaluation using morphologies, histology, and immunohistochemistry after 8 weeks of treatment showed distinct results. Morphological analysis showed that the Sham group had intact, transparent NP, while the Discectomy, GelMA, and GelMA@CINPs groups lacked NP tissue, especially the Discectomy group, which showed disorganized disc structure and dark coloration indicating severe degeneration. The NPCs and GelMA + NPCs groups exhibited small amounts of transparent tissue with relatively regular structures, while the GelMA@CINPs + NPCs group had the highest amount of transparent tissue, indicating the best repair effect (Fig. 6A).

H&E staining results were consistent with morphology (Fig. 6B), showing that at 4 weeks, histological grades increased by 2-fold, 1.9-fold, and 1.7-fold in the Discectomy, GelMA, and GelMA@CINPs groups respectively, while decreasing by 10 %, 25 %, and 42 % in the NPCs, GelMA + NPCs, and GelMA@CINPs + NPCs groups. S.O. staining for GAG deposition showed similar distribution patterns as H&E staining. Results at 4 weeks and 8 weeks did not show significant differences (Fig. 6E).

Similarly, immunohistochemistry analysis at 4 weeks showed widespread and dark collagen II distribution in the Sham group, with an average optical density of 40.5 %. In comparison, the Discectomy, GelMA, and GelMA@CINPs groups showed only 10.4 %, 10.4 %, and 15.9 % respectively. The NPCs and GelMA + NPCs groups had 14.1 % and 20.9 % respectively, while the GelMA@CINPs + NPCs group reached 29.4 %. Results at 4 weeks and 8 weeks did not show significant differences (Fig. 6F).

## 4 Discussion

IVDD is a chronic disease in which inflammatory factors and oxidative stress play crucial roles in its pathogenesis. This study aimed to construct a functional artificial NP to continuously exert antioxidant effects, thereby providing a suitable microenvironment for the growth of NPCs. While the use of GelMA for tissue engineering applications is well-documented, in this study, it merely serves as an auxiliary scaffold, while the core role is played by CINPs, which not only enhance NPC viability but also simultaneously promote ECM synthesis (e.g., upregulation of *Acan* and *Col2a1*) while suppressing matrix-degrading enzymes (*Adamts5*, *Mmp13*). This dual functionality addresses both the degenerative and regenerative aspects of IVDD. The nuclear factor kappa-B (NF-κB) pathway, a widely recognized pro-inflammatory signaling pathway, can upregulate the expression of inflammatory factors such as TNF-α [29]. The increase of these inflammatory factors in turn enhances the levels and activities of matrix metalloproteinases (MMP-9 and MMP-13), leading to the degradation of the ECM and consequently damaging the integrity of the NP [30]. Transcriptome sequencing results indicated that GelMA@CINPs could significantly downregulate the NF-κB pathway and reduce the expression of inflammatory factors such as TNF-α, demonstrating its excellent anti-inflammatory properties. Similar findings were reported by Cui et al., who developed a cuttlefish ink composite hydrogel using CINPs that reduced the levels of wound inflammatory factors and accelerated the healing of wounds infected with methicillin-resistant *Staphylococcus aureus* (MRSA)abscesses [31].

The remarkable characteristics of GelMA@CINPs are also reflected in their dual chemical-biological antioxidant properties. At the chemical level, CINPs are rich in eumelanin, whose catechol groups can act as hydrogen donors to scavenge ROS and generate quinone groups. Meanwhile, the two adjacent phenolic hydroxyl groups of the catechol groups can chelate metal ions, thereby reducing the production of ROS catalyzed by metal ions [32]. At the biological level, through experiments such as transcriptome sequencing, PCR, and WB, we found that CINPs could enhance the antioxidant capacity of cells via the NRF2/HO-1 axis. NRF2 is a transcription factor responsible for regulating the redox balance and protective antioxidant actions of mammalian cells. Under stress conditions, NRF2 is released from Kelch-like ECH-associated protein 1(KEAP1) and translocates to the nucleus, binding to the antioxidant response element (ARE)sequence and thereby promoting the transcription of *Nrf2*-regulated genes [33]. *HMOX1*, as one of the *Nrf2*-regulated genes, catalyzes the rate-limiting step in heme degradation, producing iron ions, biliverdin, and CO. Biliverdin is rapidly converted to bilirubin by biliverdin reductase (BVR), the latter of which is an effective antioxidant. For instance, Zhang et al. found that CDDO-ethyl amide can protect NPCs against high-glucose-induced oxidative injury via the NRF2/HO-1 pathway [34]. Monosaccharide analysis revealed that CINPs are rich in fucoidan, which may be the specific reason for activating the NRF2/HO-1 pathway. Fucoidan is an important component of brown algae polysaccharides, which are sulfated polysaccharides isolated from brown algae and sea cucumbers and possess excellent antioxidant properties [35]. He et al. found that brown algae polysaccharides could activate the NRF2/HO-1 pathway [36].The porous structure of GelMA is conducive to the sustained release of CINPs, and cells loaded on the microspheres can internalize CINPs into the body, thereby achieving a dual sustained release effect both in vitro and in vivo, preventing CINPs from rapidly dissipating at the IVD site and further prolonging their antioxidant action.

The promotion of NP matrix synthesis by CINPs may involve multiple pathways. CINPs are enriched in fucose, the fundamental subunit of fucoidan. As noted by Gong et al., the sulfate groups inherent to such fucose-containing structures mirror the dense negative charge of the native NP ECM [37]. This electrostatic mimicry enhances hydrogel hydration and elasticity, while the controlled release of fucose from microspheres concomitantly suppresses inflammation—collectively creating a microenvironment that indirectly drives ECM synthesis. In our study, transcriptome sequencing also revealed upregulation of ECM-receptor interaction pathways in the GelMA@CINPs group, suggesting enhanced cell-matrix interactions. Additionally, CINPs’ antioxidant effect via NRF2/HO-1 pathway reduces ROS-mediated ECM degradation and simultaneously restores anabolic capacity by up-regulating *Col2a1* and *Aggrecan* expression in NP cells [38]. Together, these mechanisms synergistically enhance ECM synthesis and promote NP regeneration. However, our study still has several limitations. First, we did not conduct an in-depth exploration of the specific endocytosis mechanism. Nevertheless, unlike inorganic nanoparticles [18], CINPs do not exhibit cytotoxicity following endocytosis. Second, we did not measure the retention time of CINPs inside the cells, making it difficult to accurately determine the precise effective time of them following the degradation of microspheres. Third, in addition to eumelanin and fucoidan, CINPs also contain other components, such as proline with antioxidant capacity, but its specific mechanism of action is not yet clear. Fourth, this experiment was limited to NPCs and did not explore the repair capacity of microspheres loaded with stem cells, which may have a more abundant array of clinical resources. Moreover, the use of rat tail vertebrae models, rather than lumbar vertebrae from large mammals such as rabbits and pigs which better approximate human intervertebral disc biomechanics, limits direct clinical translation.

## 5 Conclusion

This study has successfully developed antioxidant GelMA@CINPs microspheres to construct an artificial NP for treating IVDD. The microspheres demonstrated excellent biocompatibility and significantly enhanced the viability and proliferation of NPCs in vitro. They also promoted the synthesis of ECM and mitigated oxidative stress via the activation of the NRF2/HO-1 pathway. In vivo experiments in a rat caudal NP excision model further validated the therapeutic efficacy of these microspheres in promoting disc tissue regeneration. This work introduces a promising strategy for IVDD treatment and regenerative medicine. Future research will focus on optimizing the microsphere formulation and exploring their potential for clinical applications.

## CRediT authorship contribution statement

**Chenyang Jin:** Writing – original draft, Software, Resources, Investigation. **Jianan Chen:** Writing – original draft, Validation. **Yan Miao:** Validation. **Xin Tian:** Supervision, Resources. **Jia Wang:** Resources. **Fan He:** Supervision, Funding acquisition. **Yijie Liu:** Supervision, Resources, Funding acquisition. **Yong Xu:** Writing – review & editing, Validation, Supervision, Resources, Project administration, Funding acquisition, Conceptualization.

## Declaration of generative AI and AI-assisted technologies in the writing process

During the preparation of this work the author(s) used Deepseek in order to Polish the text. After using this tool, the authors reviewed and edited the content as needed and takes full responsibility for the content of the publication.

## Funding

This study was supported by the 10.13039/501100001809National Natural Science Foundation of China (82072476), the 10.13039/501100004608Natural Science Foundation of Jiangsu Province (BK20220046, BK20230494) and Gusu Innovation and Entrepreneur Leading Talents project (ZXL2023204), National High-level Young Talent Program (KS24700124),Jiangsu Specially Appointed Professor Program (SR24700123), the 10.13039/501100012246Priority Academic Program Development of Jiangsu Higher Education Institutions (10.13039/501100012246PAPD), The Project of 10.13039/501100002338MOE Key Laboratory of Geriatric Diseases and Immunology (No. JYN202504), Undergraduate Training Program for Innovation and Entrepreneurship, 10.13039/501100007824Soochow University (202310285164Y).

## Declaration of competing interest

The authors declare that they have no known competing financial interests or personal relationships that could have appeared to influence the work reported in this paper.

## Data Availability

Data will be made available on request.
