# Supplementary material for: Cuttlefish ink nanoparticle-engineered hydrogel microspheres synergistically attenuate disc degeneration via antioxidant defense and matrix synthesis activation
Source: Mater Today Bio. 2025 Aug 25;34:102244. doi: 10.1016/j.mtbio.2025.102244 (PMC12419125; doi:10.1016/j.mtbio.2025.102244)
Supplement: Multimedia component 1 [file mmc1.docx]

Supporting Information


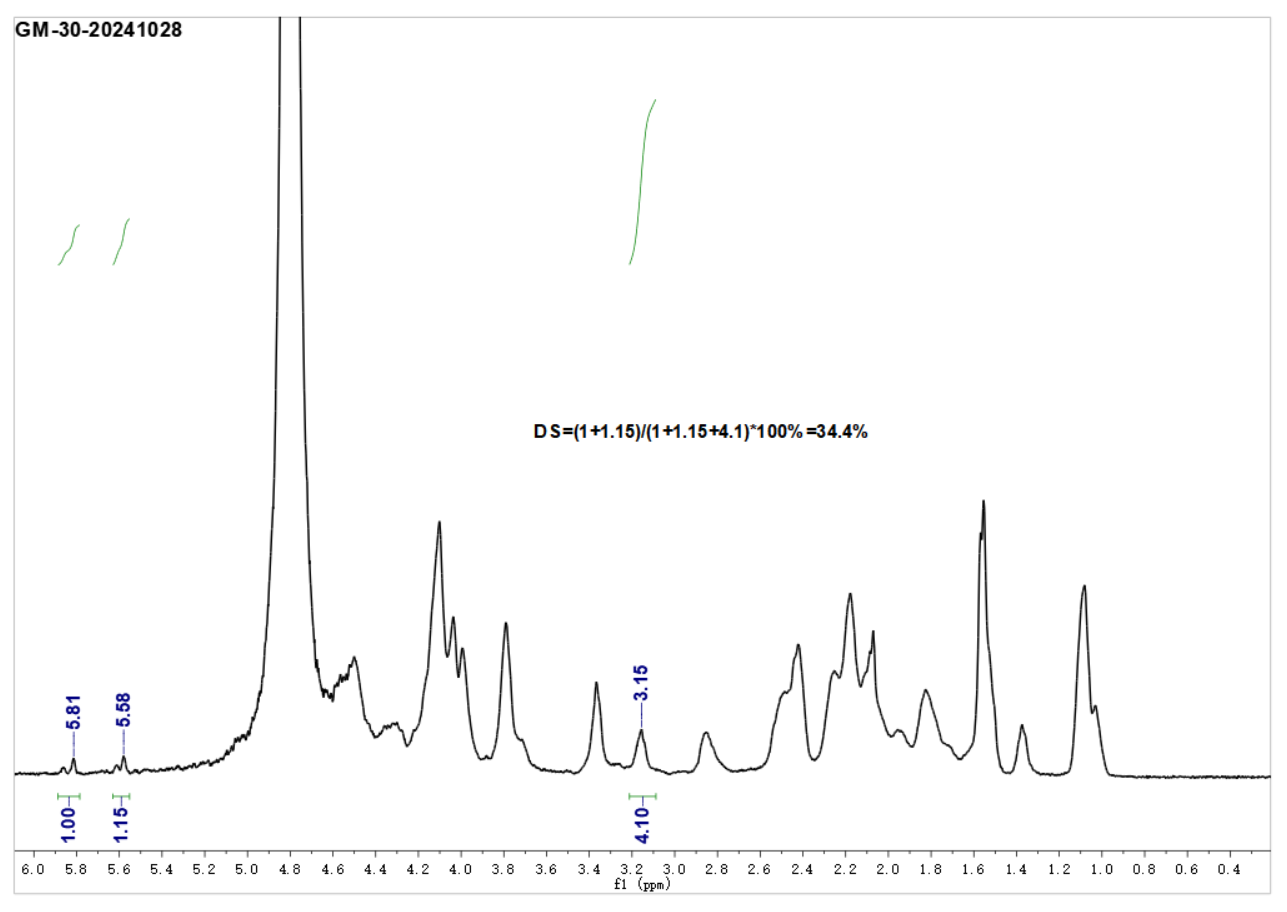


**Fig. S1.** NMR analysis of GelMA.


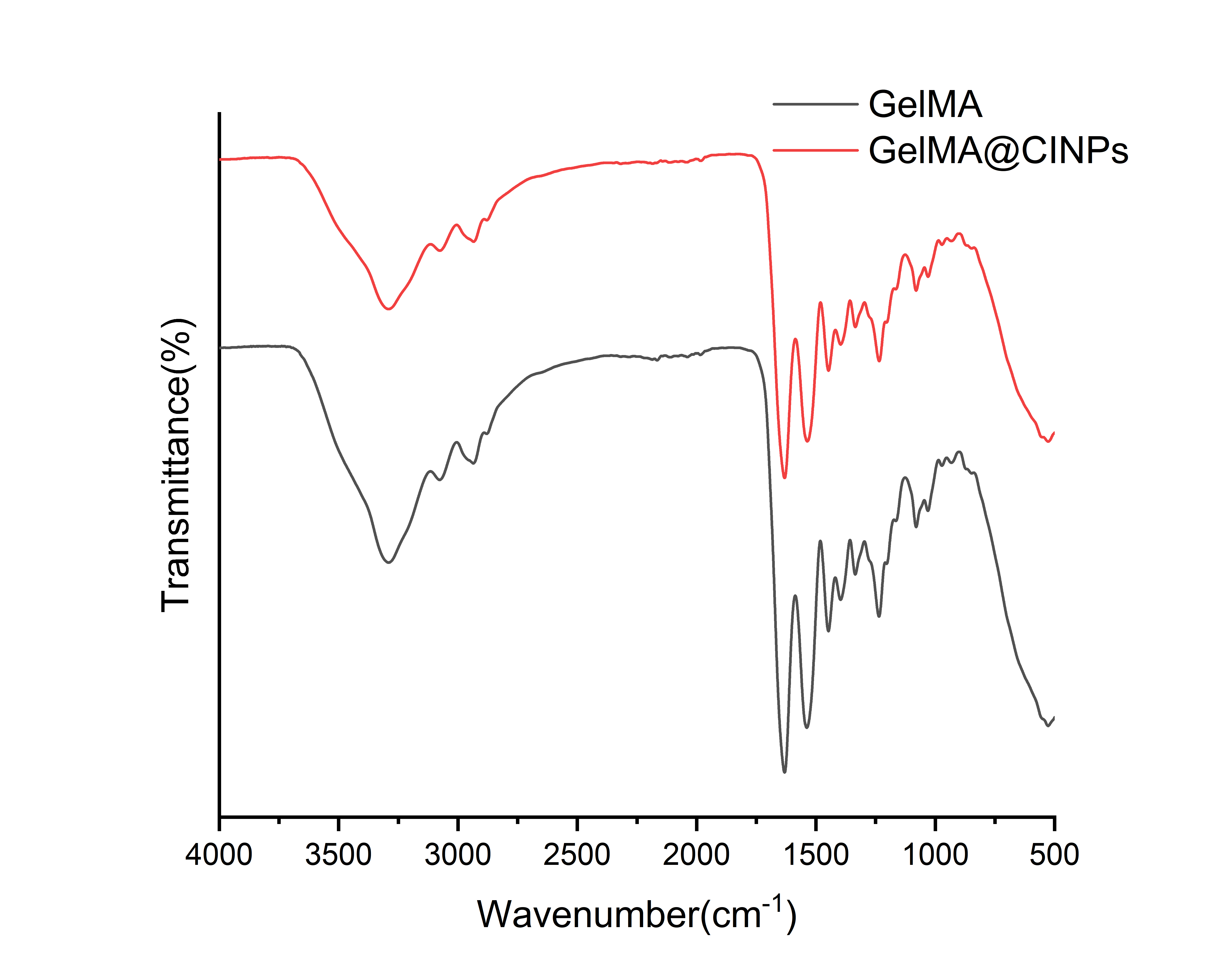
**Fig. S2.** Fourier transform infrared (FTIR) analysis of GelMA and GelMA@CINPs hydrogel microspheres.


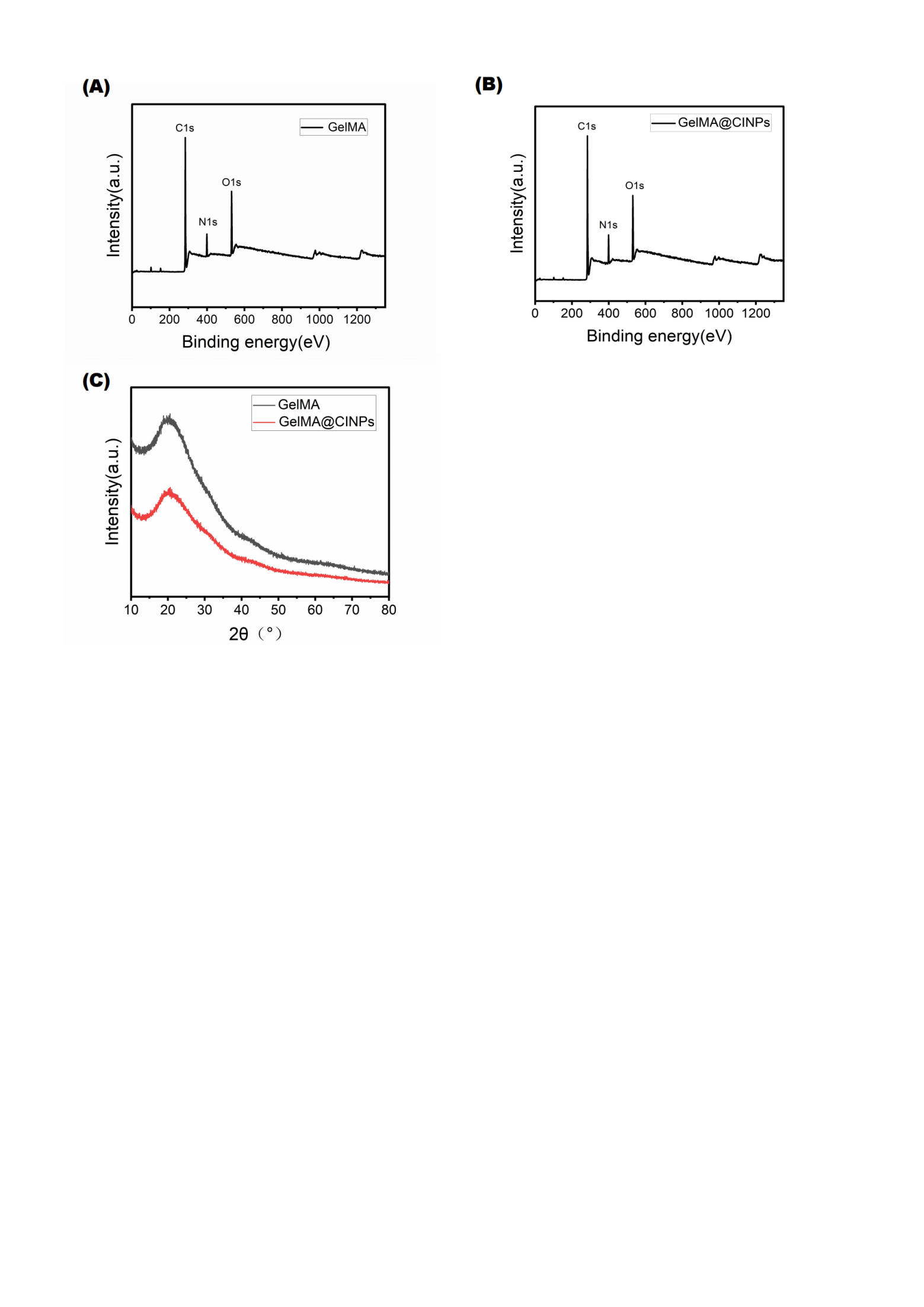


**Fig. S3.** X-ray Photoelectron Spectroscopy(XPS) and X-Ray Diffraction(XRD) of GelMA and GelMA@CINPs. A) Full XPS spectra of GelMA. B) Full XPS spectra of GelMA@CINPs. C) XRD spectra of GelMA and GelMA@CINPs.

**Fig. S4.** The mRNA expressions of matrix synthesis and degradation genes, *Acan*, *Col2a1*, *Adamts5* and *Mmp13* by RT-qPCR in NPCs.


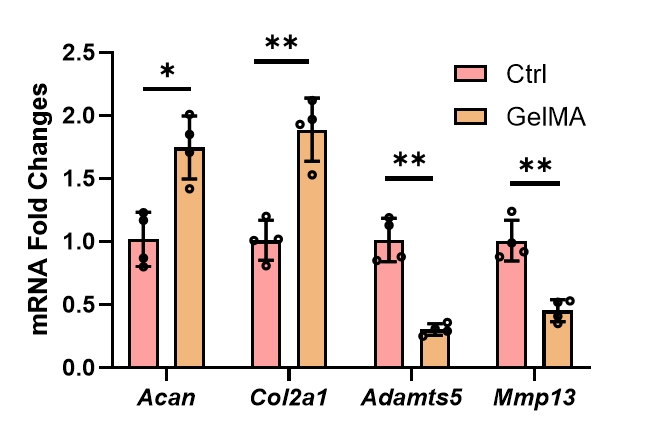


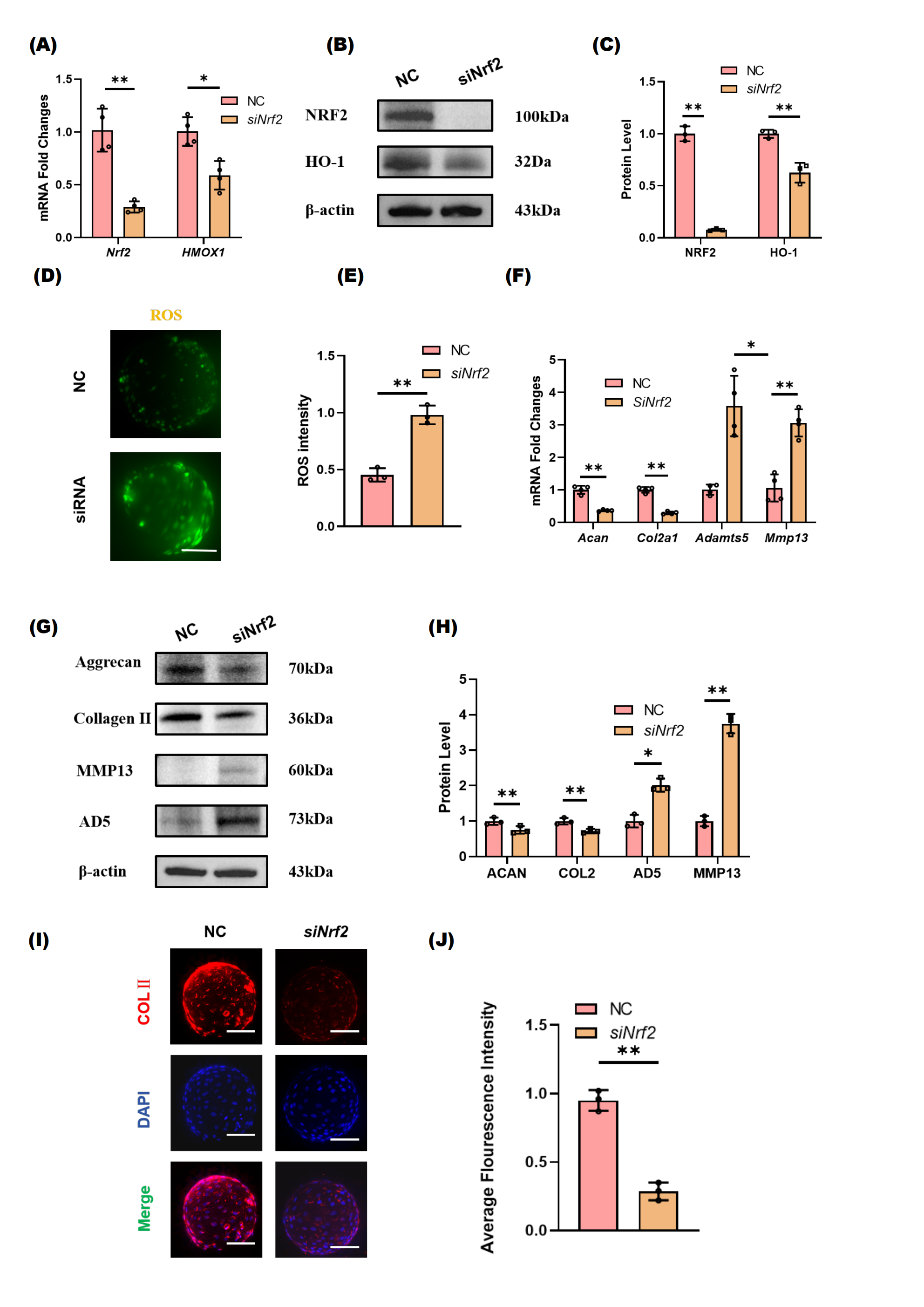


**Fig. S5.** Role of NRF2 in the functional regulation of GelMA@CINPs microsphere-cultured NPCs. A) The mRNA expressions of *Nrf2* and *HMOX1* by RT-qPCR in siRNA-transfected NPCs cultured on GelMA@CINPs. B) Western blot analysis of NRF2 and HO-1 proteins in siRNA-transfected NPCs cultured on GelMA@CINPs. C) The semi-quantitative analysis of Western blot analysis. D) The degree of intracellular reactive ROS in siRNA-transfected NPCs cultured with GelMA@CINPs was visualized through DCFH-DA-based immunofluorescence staining. Scale bar = 100 μm. E) Quantification of DCFH-DA-based immunofluorescence staining. F) The mRNA expressions of matrix synthesis and degradation genes, *Acan*, *Col2a1*, *Adamts5* and *Mmp13* by RT-qPCR in siRNA-transfected NPCs cultured on GelMA@CINPs. G) Western blot analysis of matrix synthesis and degradation proteins in siRNA-transfected NPCs cultured on GelMA@CINPs. H) The semi-quantitative analysis of Western blot analysis. I) Immunofluorescence staining of COL II in siRNA-transfected NPCs cultured on GelMA@CINPs. Scale bar = 100 μm. J) Quantification of COL II immunofluorescence staining.

**Table S1.** Primers used for quantitative RT-PCR

| **Gene** | **Forward Primer sequence (5'-3')** | **Reverse Primer sequence (5'-3')** |
| --- | --- | --- |
| *Acan* | AGGATGGCTTCCACCAGTGC | TGCGTAAAAGACCTCACCCTCC |
| *Col2a1* | CCTGGACCCCGTGGCAGAGA | CAGCCATCTGGGCTGCAAAG |
| *MMP13* | TGC TCC CAG ATG ATG ACG TT | CTG GGT CAC ACT TCT CTG GT |
| *Adamts5* | GGG AGG AGT ACA GTT TGC CT | TGA CAC TGC AGG AAC GGT AT |
| *Nrf2* | GCTATTTTCCATTCCCGAGTTAC | ATTGCTGTCCATCTCTGTCAG |
| *HMOX1* | GTAAATGCAGTGTTGGCCCC | ATGTGCCAGGCATCTCCTTC |
| *Gapdh* | GCAAGTTCAACGGCACAG | CGCCAGTAGACTCCACGAC |

**Table S2.** Antibodies used in Western blot.

| **Primary Abs** | **Dilution** | **Cat No.** | **Company** |
| --- | --- | --- | --- |
| Aggrecan | 1 : 500 | FNab00213 | FineTest |
| Collagen II | 1 : 2,000 | ab188570 | Abcam |
| MMP13 | 1 : 2,000 | A11148 | Abclonal |
| ADAMTS5 | 1 : 2,000 | A23125 | Abclonal |
| NRF2 | 1 : 2,000 | A21176 | Abclonal |
| HO-1 | 1 : 2,000 | A1346 | Abclonal |
| β-actin | 1 : 2,000 | AF5003 | Beyotime |
| **Secondary Abs** |  |  |  |
| goat anti-rabbit horseradish peroxidase (HRP)-conjugated | 1 : 20,000 | ab6721 | Abcam |
